# Systematic review of co-occurring transition of retirement and migration: An occupational perspective

**DOI:** 10.1177/03080226251335450

**Published:** 2025-05-19

**Authors:** Michael Haan, Jackie Eagers, Ylona Chun Tie, Fiona Barnett, Hans Jonsson, Sirirat Seng-iad

**Affiliations:** 1 College of Healthcare Sciences, James Cook University, Townsville, QLD, Australia; 2 Occupational Therapy and Occupational Science, Karolinska Institutet, Stockholm, Sweden; 3 Sirindhorn School of Prosthetics and Orthotics, Faculty of Medicine Siriraj Hospital, Mahidol University, Bangkok, Thailand

**Keywords:** Retirement migration, occupational transitions, occupational adaptation

## Abstract

**Introduction:**

Retirement signifies a major life transition, affecting daily structure, identity and social connections. Migration in retirement is a growing societal phenomenon with significant implications for daily life, affecting retirees’ occupations, identity, cultural adaptation, social networks and well-being. This review explored how international retirement migration is represented in the literature, focusing on motivations, its dual role as retirement and migration, and its examination through an occupational lens.

**Methods:**

Six databases were searched for relevant studies on retirement migration, resulting in the inclusion of 13 studies. Reflective thematic analysis was used for coding, leading to the identification of three major themes representing retirees’ experiences.

**Results:**

Three broad overarching themes were identified from the thematic analysis: (i) Factors in decision-making for retirement migration, (ii) Healthcare and well-being in retirement migration and (iii) Settling in the retirement country.

**Conclusion:**

Permanent international retirement migration is driven by factors including lower living costs and favourable climates and significantly impacts retirees’ economic conditions, integration and well-being. This review contributes insights into the occupational, social and well-being aspects of retirement migration, emphasising the importance of community integration and calling for further research on international retirement migration from an occupational perspective.

## Introduction

Retirement typically signifies the end of a long-term occupation for those who have worked most of their lives. Besides financial benefits, work offers daily structure, meaningful engagement, a sense of identity and social connections (Eagers et al., 2019). The work-to-retirement transition is a significant life change with implications for daily occupations (Jonsson et al., 2000; Pettican and Prior, 2011). Retirement from work is an occupational transition where retirement often leads to less physical activity and increased sedentary time (Suorsa et al., 2022). Most people can adjust to this slower pace of life, but retirement can also bring unforeseen challenges and frustrations (Jonsson et al., 2001). The loss of a work routine can lead to a more unstructured life and lead to feelings of loneliness or loss of identity. Some retirees might choose to continue working to some degree, volunteer with a charity, organisation, or club, or decide to cease working entirely (King et al., 2021). Kauppi et al. (2021) found that retirees’ social circle can diminish during the retirement transition, this is attributed to the loss of work-related contacts. Retirees must maintain and expand their social networks and structure their daily lives, as actively managing daily routines and occupations is key to a positive retirement experience (Vigezzi et al., 2021).

Retirement migration is a worldwide societal phenomenon defined as the voluntary relocation of individuals or couples to other countries (King, 2021). Determining the exact number of people who migrate upon starting their retirement is challenging. According to the Office for National Statistics in the United Kingdom (UK), around 247,000 British retirees were living in other European countries in 2017 (Office for National Statistics, 2017). The US State Department reports that migration in retirement has significantly risen – suggesting up to five million Americans, or 12% of American retirees, live abroad (Harvey Law Group, 2023). An increase in retirement migration signifies the need to understand this complex phenomenon from different perspectives.

Migration, like retirement, is a life-changing event with significant implications for daily life (Gupta and Hocking, 2018). Migration affects retirees’ occupations, identity, cultural adaptation, social networks and overall well-being (Bennett et al., 2012). As life expectancy increases, retirees are spending more time in retirement, which poses financial challenges if savings must potentially support a more extended period without additional income from work (Savaş et al., 2023). Moreover, globalisation and cheaper travel options may influence retirees’ perspectives to explore retirement options in countries with better healthcare, climate and lifestyle opportunities (Savaş et al., 2023).

Retirement migration combines two occupational transitions: retirement and migration. Each requires its own set of adjustments and impacts on role, identity and social life. When retirement and migration co-occur, they influence each other and are intertwined (Haan, 2025). Occupational science and therapists view ‘occupation’ as a complex concept involving all the activities, roles and tasks individuals engage in throughout their lives (Hinojosa and Kramer, 1997). This occupational perspective includes work but also activities related to self-care, productivity, leisure and social participation. Occupational science explores how various occupations contribute to an individual’s overall well-being, identity and quality of life (Crepeau et al., 2003). Given occupational therapy’s focus on occupation and the changes in an occupation that occur due to retirement and migration, the profession is well-placed to explore and provide support within this transition. By exploring how retirement migration affects daily activities, social connections and well-being, highlighting opportunities for further research and ways occupational therapy can support retirees in adapting to their new communities.

This review had three main aims: first, to uncover the current body of knowledge on the co-occurring transition of retirement migration, including the factors and motives underpinning the decision-making process of people planning their retirement abroad. Second, what has been described regarding the interplay of retirement and migration as two occupational transitions, and thirdly, what is known about international retirement migration from an occupational perspective. The research question for this literature review was: what is currently known about permanent international retirement migration as a co-occurring occupational transition?

## Method

### Search strategy and selection criteria

The systematic review protocol for this study has been registered with PROSPERO under the ID CRD42024417808 for the year 2024. Databases and search terms were selected in consultation with the faculty librarian. Initial searches were conducted by the first author and discussed with the second and last author. Through reading a number of full-text studies, key concepts were identified for this literature review: retirement, migration and co-occurring transitions. Retirement was defined as the complete cessation of paid employment and migration needed to be both permanent and international. In addition, we determined that migration should be a deliberate part of retirement planning and voluntary; thus, participants had to experience both the retirement and migration transitions simultaneously. This led to excluding terms such as ‘snowbird,’ ‘seasonal,’ ‘grey nomads,’ ‘turn around migration’ and ‘return migration,’ as they signify either temporary migration or a return to the home country due to visa implications.

**Figure 1.**
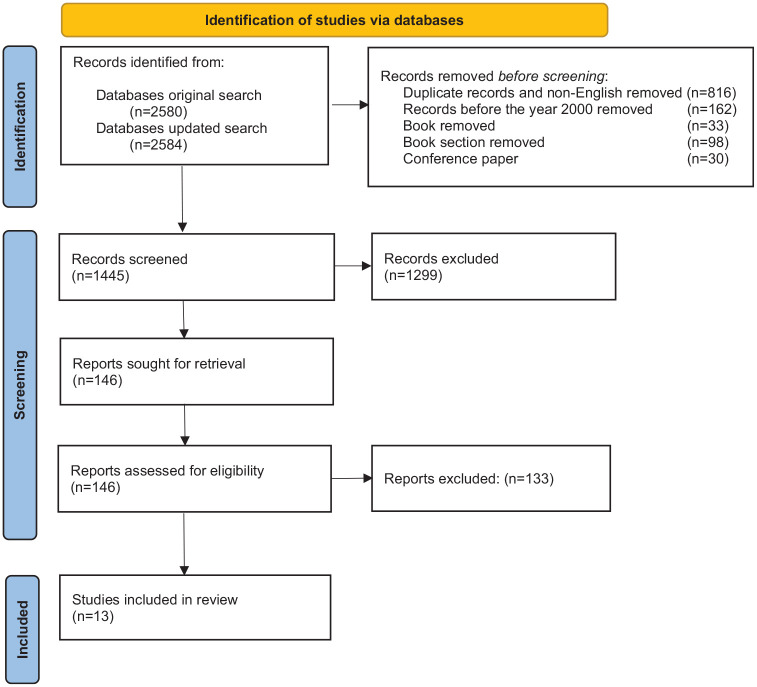
PRISMA flow diagram. Source: Moher et al. (2009).

The search was conducted in July 2023 and updated in August 2024 by the first author. PRISMA (Page et al., 2021) flow chart represents the steps to determine the included studies (Figure 1). Six databases, including CINAHL, Emcare, SCOPUS, MEDLINE, Emerald Insight and ScienceDirect, were systematically searched using predefined search terms: Retirement, Retire*, Retiree, Retirees, ‘Leisure Retirement’, Migration, Migrant*, ‘International Retirement’, ‘Retirement Migrations’, ‘Retirement Immigrations’, ‘International lifestyle’, ‘Tourism-led migration’.

### Inclusion criteria

- All study designs are considered for inclusion, provided they specifically focus on retirement migration.
- Participant retirement status: Study participants should be fully retired, meaning they have entirely ceased paid work.
- Permanent and international migration: The studies should involve participants who have undergone permanent and international migration.
- Investigation of migrated retirees’ experiences: The studies should specifically investigate the experiences, motivations and challenges faced by migrated retirees.
- Language requirement: All studies included in the review should have been written in English.
- Publication criteria: The studies must be published in peer-reviewed journals and should be original research.
- Time frame: Studies should have been published from 2000 onwards.

### Selection process

Duplicates and book chapters were removed, and the remaining study titles were assessed for eligibility using established criteria. The research team reviewed the study abstracts and discussed any uncertainties. Subsequently, the first and the sixth author independently assessed the full texts of 146 identified studies. Three further studies were identified for inclusion when the search was rerun. Discrepancies in their assessments were resolved through collaborative discussions.

### Quality assessment

The included studies were appraised for their methodological quality using the McMaster Guidelines and Appraisal Forms for Qualitative Research (Letts et al., 2007). To illustrate the methodological quality of the studies, a numerical score was added to the McMaster appraisal forms as used by other studies (Alexandratos et al., 2012), with a modification by adding scoring for reporting on consent from the participants. The first and the second authors independently conducted this quality assessment for the first two studies and later randomly for another three. Any discrepancies or uncertainties were reviewed and resolved by consensus.

### Data extraction and analysis

Data extraction focused on participant details, countries of retiree migration, study purpose, design, participant ages and key findings. This involved extracting narratives and experiences from study results. The first author conducted this process, with subsequent checks and discussions with the research team.

The data was coded using reflective thematic analysis (Braun and Clark, 2019). The first author completed the initial coding. These codes identified initial themes, which were refined through comparison with the data and discussion with team members, leading to more nuanced themes that accurately represented migrated retirees’ experiences.

## Results

One hundred and forty-six studies were appraised for relevance, resulting in a final dataset of 13 studies included in the systematic review (Table 1). All included studies had a qualitative study design; three were conducted using an ethnographic study design, one had a phenomenological approach, one had a narrative approach, one study used grounded theory, and the other seven studies did not indicate a specific qualitative design. McMaster Critical Appraisal scores (out of 24) ranged from 10 to 19. Although both qualitative and quantitative studies were screened at the full-text level, only qualitative studies met the inclusion criteria.

**Table 1.** Description of included studies.

Description	Home country	Retirement country	Study purpose	Study identified finding	McMaster critical appraisal score out of 24
*Ashton et al. (2019) Place development for international retirement migrants: A decision-making process model. Journal of Place Management and Development*					
*n* = 33 (27 male, 6 female) Age 55–79	Germany (8), the USA (7), France (6), Australia (5), Austria (2), Sweden (2), Norway (1), Switzerland (1) and UK (1)	Thailand	This study investigates the decision-making processes of international retirement migrants.	Future retirees can choose a place to move based on their feelings and the connections they formed during earlier visits. Understanding how people decide to stay for a short time, for a longer but not permanent period, or to move permanently is crucial for those managing destinations.	15
*Haas (2013) Volunteering in retirement migration: Meanings and functions of charitable activities for older British residents in Spain. Ageing and Society*					
*n* = 24 (9 male, 15 female) Age 60+	Belgium, the Netherlands, Austria, France, Switzerland, Germany, South Africa, the USA and Australia *(numbers not available)*	Spain	Examining the role of volunteering during retirement migration in managing health, fostering adaptation and serving as a cultural expression.	The British people living on the Costa Blanca who volunteer and help with charities play a big part in dealing with health issues faced by older people who have moved there. This also shows that the British community has strong social bonds and supports each other well.	11
*Hayes (2021) ‘Sometimes you gotta get out of your comfort zone’: Retirement migration and active ageing in Cuenca, Ecuador. Ageing and Society*					
*n* = 83 (male/female numbers not identified) Age 54–80, however, age was not recorded for the complete sample	Canada and the USA *(numbers not available)*	Ecuador	This study looks into how the ideas of limited time and the fears of a final stage in a life filled with decline and death affect the choices and dreams of Canadian and American people who move to another place for retirement.	This study shows how retirement narratives intertwine with cultural shifts, showcasing the evolution from a post-war consensus to a neoliberal era, where ideals of healthy ageing and adventure-seeking among international retirement migrants reflect both individual responsibility and societal transitions in changing economic landscapes.	10
*Innes (2008) Growing older in Malta: Experiences of British retirees’. International Journal of Ageing and Later Life.*					
*n* = 16 (8 male, 8 female) Age 55–80	UK	Malta	This research focuses on understanding the experiences of people who retire from the UK and then move to Malta to live.	This study emphasises how societal influences affect retirees who choose Malta for retirement, focusing on factors such as climate, lifestyle, cost of living and familiarities such as their home countries.	17
*Kohno et al. (2016) Issues in healthcare services in Malaysia as experienced by Japanese retirees’. BMC Health Services Research*					
*n* = 30 (14 male,16 female) Age 54–79	Japan	Malaysia	This study looks into the important problems related to healthcare services that Japanese retirees face in this country.	Japanese retirees face four key healthcare challenges: ‘language barriers’, ‘trust and distrust in healthcare services’, ‘information shared through personal stories’ and issues related to ‘nursing and end-of-life care’.	16
*Lardiés-Bosque et al. (2015) Retirement migration and transnationalism in northern Mexico. Journal of Ethnic and Migration Studies*					
*n* = 29 (male 15, female 14) Age 55–64 (*n* = 11), 65–74 (*n* = 14), 75 or older (*n* = 4)	The USA	Mexico	This article looks into the trend of living in more than one country among retired people who have moved to the border areas of northern Mexico.	This study shows that viewing migration through a transnational lens offers significant benefits compared to traditional views, which only consider migration from the perspective of the country receiving immigrants.	15
*Marquez et al. (2024) Americans in Pursuit of an Age-Friendly Retirement Haven in Mexico. Activities, Adaptation & Aging*					
*n* = 20 (12 male, 8 female) Age 62–84 (mean 72)	The USA	Mexico	The research aims to explore the lived experiences of American retirees in Mexico and examine the challenges and benefits of their retirement migration.	Retirees were generally satisfied with their decision to migrate, citing a welcoming local culture, affordable lifestyle, quality healthcare and opportunities for social engagement and physical activity as primary benefits.	16
*Repetti et al. (2024) Developing ‘Age-Friendly’ Communities: The Experience of International Retired Migrants. Sociological Research Online*					
*n* = 87 (male 36, female 51) Age 47–83 (mean 66)	UK, Switzerland, France, the USA and Canada *(numbers not available)*	Spain, Costa Rica and Mexico	To understand the experiences of retired migrants and the factors that contribute to creating age-friendly communities in their new environments.	The themes identified include the sense of normalcy, the economic value of older people and the social support networks within expatriate communities. The study highlights the advantages of living in an age-friendly community and the importance of belonging and social inclusion for retirees.	18
*Repetti and Calasanti (2020) Retirement migration and transnational grandparental support: A Spanish case study. Global Networks*					
*n* = 27 (9 male, 18 female) Age 56–83 (mean = 67)	UK (12), Switzerland (9), France (3), Belgium (2), Germany (1)	Spain	This study explores how retirees who migrate navigate grandparental support dynamics.	The findings reveal that despite geographical distance, these retirees remain actively engaged in providing care, reshaping the meaning of grandparental support and perceiving their familial relationships as deeper. This demonstrates the nuanced impact of migration on caregiving roles and the quality of familial connections.	16
*Repetti and Calasanti (2020) The cultural and structural motivations of cheap mobility: The case of retirement migrants in Spain and Costa Rica. Geoforum*					
*n* = 59 (25 male, 34 female) Age 47–83 (mean = 62)	The USA (25), UK (13), Switzerland (11), France (3), Belgium (3), Canada (2), Germany (1) and the Netherlands (1).	Spain	This article explores why retired individuals from wealthier countries choose lifestyles that involve frequent travel.	This study found that retirement migrants are reliant on cheap air travel for economic security and family support and show how aero mobility becomes crucial for retirees experiencing economic uncertainty and serves as a means to maintain connections with family members abroad.	12
*Repetti et al. (2018) Retirement migration in Europe: A choice for a better life? Sociological Research Online*					
*n* = 29 (10 male, 19 female) Age 51–83 (mean = 66)	UK (14), Switzerland (8), Germany (1), Belgium (2), France (3), The Netherlands (1)	Spain	This article explores how economic differences affect retirees from Northern Europe’s decisions when they move to countries in the Mediterranean.	For many retirees moving to new countries, the decision to migrate is a strategy to handle economic uncertainties, aiming to better their financial conditions and social standing. However, this move also brings about new challenges and risks.	19
*Sloane et al. (2013) Healthcare experiences of U.S. Retirees living in Mexico and Panama: A qualitative study. BMC Health Services Research*					
*n* = 46 (23 male, 23 female) Age 59–87	The USA	Mexico (23) Panama (23)	This study describes the healthcare experiences and perceptions of retired U.S. citizens in Mexico and Panama, including challenges and satisfaction with local healthcare systems.	Retirees appreciated personalised physician services, affordable home care and quality outpatient and dental care. However, concerns were raised about the unavailability of Medicare, reduced Tricare coverage and challenges in accessing emergency services and specialised procedures.	18
*Toyota (2022) Shut-In Abroad: Social incapacitation among low-income male Japanese retirees in Thailand. American Behavioral Scientist*					
*n* = 48 male Age 50–87	Japan	Thailand	To study how single male retirees from Japan are moving to Southeast Asia to live alone because they feel ashamed and disconnected in Japan.	The research shows that the men felt socially unable to connect due to work, family and societal expectations in Japan. It emphasises the importance of gender norms and emotions in ageing and how past experiences shape future perspectives.	11

The 13 included studies collectively represented participants from over 20 countries. Countries from which retirees moved include the USA, Australia, Austria, Belgium, Canada, France, Germany, Japan, the Netherlands, Norway, South Africa, Sweden, Switzerland and the United Kingdom. Countries retirees migrated to include Costa Rica, Ecuador, Malaysia, Malta, Mexico, Panama, Spain and Thailand.

## Themes and key findings

Thirteen articles, all qualitative, were included in this systematic literature review. Three broad overarching themes were identified from the thematic analysis: (i) Factors in decision-making for retirement migration, (ii) Healthcare and well-being in retirement migration and (iii) Settling in the retirement country. The review did not identify studies that explored the connection between retirement and migration as two occupational transitions. Similarly, none of the included studies looked at international retirement migration from an occupational perspective.

### Factors in decision-making for retirement migration

The studies identified that people’s decisions to migrate on retirement were multifactorial.

Seven studies explored the objectives and goals that were achieved by choosing to migrate for retirement, and what factors contributed to making this decision (Ashton et al., 2019; Hayes, 2021; Innes, 2008; Marquez et al., 2024; Repetti and Calasanti, 2020; Repetti and Lawrence, 2021; Toyota, 2022). Participants reflected on what was realised regarding their quality of life by migrating. The migration was due to financial and climate conditions that would support a healthy retirement lifestyle. Retirees assessed their decision to move by comparing their current life with their expectations if they had not migrated.

Several studies examined the decision-making process involved in retirement migration. Ashton et al. (2019) focused on the initial awareness and interest in deciding on migration and potential retirement locations. The authors found that individuals were introduced to the idea of retirement migration by friends or family who had previously chosen this path. Furthermore, some participants had holiday experiences in the retirement country where there was no prior intention to migrate for retirement but gradually warmed up to the idea over time (Ashton et al., 2019). Hayes (2021) highlighted how caregiving responsibilities towards elderly parents hindered the formulation of concrete retirement migration plans. Ironically, being surrounded by ageing and ill individuals subsequently became one of the reasons to choose retirement migration (Hayes, 2021). In contrast, Repetti and Calasanti’s (2020) study illustrated how retirement migration had created new opportunities, providing caregiving roles for grandchildren. This migration enabled retirees to host their grandchildren during the summer holidays and spend more time with them than they normally would have (Repetti and Calasanti, 2020).

The reasons for retirement migration from the studies were economic factors and a climate that encouraged the expected retirement lifestyle. Retirees reflected on the opportunities gained from comparing their current life with a life in a migration destination (Ashton et al., 2019; Hayes, 2021; Innes, 2008; Marquez et al., 2024; Repetti and Lawrence, 2021; Toyota, 2022). When considering retirement migration, the economic situation of the individuals was important as participants sought a lower cost of living to stretch their retirement funds (Ashton et al., 2019; Hayes, 2021; Marquez et al., 2024; Repetti et al., 2018). The reduced daily costs allowed retirees to engage in activities that might have been unaffordable had they stayed in their country of origin. Examples given by participants were dining out in restaurants, going out to bars or café, local travel and engaging in sports activities (Ashton et al., 2019; Hayes, 2021; Marquez et al., 2024; Repetti et al., 2018). Studies also mentioned the retiree’s ability to afford to take dancing and language classes (Hayes, 2021) and join cultural events (Repetti et al., 2018). Due to the better climate, participating in outdoor activities, including going to the beach or playing golf, was possible (Ashton et al., 2019). In their new lifestyle, participants found increased social engagement options (Haas, 2013), a more scenic environment that they enjoyed (Ashton et al., 2019), and a sense of adventure (Hayes, 2021).

### Healthcare and well-being in retirement migration

Seven studies identified how healthcare and well-being were significant factors in retirement migration. Four studies explored retirees’ views on healthcare after migration. They explored how retirees made healthcare decisions, managed their health conditions and accessed healthcare in their retirement country (Haas, 2013; Kohno et al., 2016; Repetti et al., 2018; Sloane et al., 2013). Two studies mentioned retirees’ satisfaction with healthcare services in Spain and Malaysia and their trust in medical staff (Haas, 2013; Kohno et al., 2016). British migrated retirees found the healthcare in Spain superior to that in the United Kingdom (Haas, 2013). Japanese migrated retirees in Malaysia and American retirees in Mexico had some concerns about the standard of healthcare in their retirement country (Kohno et al., 2016; Sloane et al., 2013). In two other studies, participants mentioned that the healthcare they received in the retirement country was more personal and affordable compared to their origin country (Marquez et al. 2024; Sloane et al., 2013). One study also highlighted the concern of migrated retirees about their access to healthcare after Brexit (Repetti et al., 2018). When participants received first-hand information from other migrated retirees the likelihood of receiving medical care in the retirement country increased (Repetti et al., 2018). Accessing healthcare in the host country involved overcoming trust issues, language barriers and finding affordable healthcare insurance (Haas, 2013; Kohno et al., 2016; Repetti et al., 2018; Sloane et al., 2013).

In general, migrated retirees mentioned the improved opportunities to maintain their health and well-being after migrating. Five studies investigated factors contributing to retirees’ overall physical and mental well-being (Ashton et al., 2019; Haas, 2013; Hayes, 2021; Marquez et al., 2024; Repetti et al., 2018). Although these studies did not explicitly mention physical or mental well-being, several studies discussed aspects related to well-being. For example, by migrating, participants gained opportunities for an active lifestyle and being more outdoors, which might not be possible in their home country (Ashton et al., 2019; Hayes, 2021; Marquez et al., 2024). As part of their well-being, participants also mentioned the interactions with friendly locals (Ashton et al., 2019) and engagement in social activities (Ashton et al., 2019; Hayes, 2021; Marquez et al., 2024; Repetti et al., 2018). In addition, participants also mentioned a sense of adventure and excitement in having all these new experiences after migrating, contributing to their satisfaction and well-being (Hayes, 2021). Retirees who migrated described in several studies how they have increased opportunities to manage their well-being in the retirement country (Ashton et al., 2019; Haas, 2013; Hayes, 2021; Marquez et al., 2024; Repetti et al., 2018).

### Settling in the retirement country

Nine studies identified how participants adapted to their retirement country after the initial period of their migration (Haas, 2013; Innes, 2008; Kohno et al., 2016; Lardiés-Bosque et al., 2015; Marquez et al., 2024; Repetti and Calasanti, 2020; Repetti et al., 2024; Repetti and Lawrence, 2021; Toyota, 2022). These retirees navigated new communities, language barriers, family life and social interactions in the new environment.

Engaging in local organisations significantly helped with getting established in the new context. Examples given by participants were volunteering or meeting with other migrated retirees, especially those from their origin country (Haas, 2013; Innes, 2008; Lardiés-Bosque et al., 2016; Marquez et al., 2024; Repetti et al., 2024). This engagement initially came from a desire among migrated retirees to contribute positively to their host communities, and it enhanced their sense of community and belonging (Haas, 2013; Innes, 2008; Marquez et al., 2024; Repetti et al., 2024). One participant mentioned transitioning from activities associated with a holiday experience that revolves around leisure, eating and drinking to finding more meaning and structure by engaging in a charity organisation (Haas, 2013). Furthermore, migrated retirees commonly liked to be perceived as active members of their new communities rather than as outsiders; this also contributed to engagement in local organisations (Ashton et al., 2019; Repetti et al., 2024).

Migrated retirees in four studies mentioned that they had difficulties speaking and understanding the language of the country they migrated to (Haas, 2013; Kohno et al., 2016; Lardiés-Bosque et al., 2015; Repetti et al., 2024). These retirees wanted to connect, communicate and be part of their new communities, but the language barrier made this challenging. In addition, these participants primarily socialised with other migrated retirees from their country of origin (Haas, 2013; Kohno et al., 2016; Lardiés-Bosque et al., 2015). Some participants in the study of Marquez et al. (2024) mentioned that they found comfort in not needing to learn the local language due to the large English-speaking community. Participants reported having better social lives in their new communities than they would have had if they had stayed in their home countries (Repetti et al., 2024).

Safety emerged as a concern in retirement migration, with two studies (Ashton et al., 2019; Innes, 2008) mentioning that participants felt safer in their retirement country. Retirees in Thailand perceive the country as safe and peaceful, appreciating its culture and respect for elders and feeling more secure there than in their home countries (Ashton et al., 2019; Innes, 2008; Marquez et al., 2024). Migrated retirees compared this feeling of safety with their concerns about the situation in their country of origin. These concerns included worries about terrorism, overcrowding and general insecurity (Ashton et al., 2019). British retirees in Malta feel safer than in the UK and believe that the secure environment in Malta has improved their quality of life and opportunities for social engagement (Innes, 2008).

Furthermore, the studies reported on settling into the new context and focused on how migrated retirees navigated existing family ties (Innes, 2008; Repetti and Lawrence, 2021; Toyota, 2022). This involved travelling back to their home country and receiving family members in the retirement country (Innes, 2008; Repetti and Calasanti, 2020; Repetti and Lawrence, 2021; Toyota, 2022). Although participants in one study expressed concerns about their ability to travel back and forth, they generally reflected on the positive aspects of these new family dynamics (Innes, 2008; Repetti and Calasanti, 2020; Repetti and Lawrence, 2021; Toyota, 2022). Many considered their retirement migration successful, emphasising the positive experiences, while some faced challenges maintaining family ties (Repetti and Lawrence, 2021). On the contrary, other studies highlighted how retirement migration had led to closer family relationships (Repetti and Calasanti, 2020; Toyota, 2022). This closeness resulted from spending more quality time together when the family visited the retirees in the retirement countries (Innes, 2008; Repetti and Calasanti, 2020; Repetti and Lawrence, 2021; Toyota, 2022).

## Discussion and implications

This review provided an overview of peer-reviewed work on permanent international retirement migration. Findings explored the current body of knowledge on the co-occurring occupational transitions of retirement migration, specifically factors and motives in the decision-making process of planning retirement abroad and the interplay of retirement and migration as occupational transitions. Three themes were identified based on 13 qualitative studies: (i) Factors in decision-making for retirement migration, (ii) Healthcare and well-being in retirement migration and (iii) Settling in the retirement country. These themes can be looked at as a process retirees go through, from their reasons for moving to the adjustments they make as they settle into a new country. This process reflects how retirees navigate the decision to migrate, adapt to a new environment, establish new routines and integrate socially, all while prioritising their health and well-being as essential elements throughout their migration journey.

As individuals approach retirement, they reflect on their current living situation and assess how well it aligns with their expectations and aspirations for a fulfilling retirement. Expectations of having an active and healthy retirement influenced participants’ decision to migrate. Important considerations were financial and climate conditions. The findings tie well with prior studies on retirement migration, namely that climate often drives seasonal retirement migration (Kou et al., 2018). Retirees in the included studies migrated primarily for affordability and economic benefits, leading to an improved lifestyle with more opportunities for occupational engagement. Many retirees positively compared their new lifestyle to what they would have had if they had not migrated, highlighting a desire for active, warm and socially engaging environments, which are often found in popular tropical destinations in Asia (Ashton et al., 2019; Kohno et al., 2016; Marquez et al., 2024; Toyota, 2022). Moving to a new country often requires people to take on new roles and routines, which can help them adjust to life after work. A recent study found that retirees who moved to Thailand often became involved in new activities, like helping local communities or taking care of their health, which gave structure and meaning to their retirement (Haan, et al., 2025).

Retirees reflected on how their lives have improved after migrating (Hayes, 2021; Innes, 2008; Repetti and Calasanti, 2020; Repetti and Lawrence, 2021; Repetti et al., 2018; Toyota, 2022) as their new context is offering more opportunities for occupational engagement. This reflection aligns with Özyurt’s (2023) findings, which indicate that migration not only enhances retirees’ lifestyles but also promotes personal growth, positive relationships and immersion in a welcoming host culture. By migrating, some retirees experience closer relationships with family, while some do not due to the distance and expenses of travelling. As individuals retire, they experience a decrease in the activities that structure their daily lives and provide meaning, including opportunities for social engagement (Suorsa et al., 2022). It seems that for retirees who choose to migrate, the migration transition itself becomes a new adventure and challenge in life. This is in line with earlier research about the importance of finding engaging occupations in retirement (Jonsson et al., 2000). Migration offers retirees unique opportunities for occupational engagement by exploring new roles, developing skills in adapting to different cultures and engaging in various activities that enhance their well-being.

Migration offers retirees numerous opportunities for occupational engagement, as it is filled with new adventures (Hayes, 2021) and activities (Ashton et al., 2019). The occupational transition in migration encompasses everything from planning and organising to adapting to new environments and engaging in diverse social and cultural activities. Given the expertise of occupational therapists in facilitating occupational engagement and adapting to new environments, it is important for occupational therapists working with retirees that migrated to understand these engagements. This understanding equips occupational therapists to effectively address the unique challenges and opportunities faced by migrated retirees. An occupational perspective is not only academically important; it also carries practical implications in enhancing therapists’ abilities to facilitate successful co-occurring occupational transitions in retirement migration.

Some retirees viewed migration as the only way to ensure a comfortable retirement due to economic constraints (Marquez et al., 2024; Repetti and Lawrence, 2021), while some faced a care gap in challenging situations such as health decline or loss of a partner (Sampaio, 2017). Therefore, while retirement migration can offer a higher standard of living in warmer climates and potentially closer family bonds, it also underscores the complex balance between economic advances and the potential for increased isolation and vulnerability, emphasising the need for a nuanced understanding of retirement migration’s impact.

The findings suggest that being part of a community is essential in settling in the retirement country. In the occupational transitions of retirement migration, an important aspect of the existing literature reinforces the findings from this study that retirees want to actively participate in organisations and integrate into their retirement countries’ communities (Haas, 2013). From an occupational perspective, this participation is seen as a crucial aspect of maintaining a sense of purpose and belonging (Haim-Litevsky et al., 2023), which is important for overall well-being in later life. Doing things such as community work, volunteering, or cultural activities brings a sense of belonging and helps with physical health. According to the occupational adaptation framework (Schkade and Schultz, 1992), such activities facilitate an adaptive response to new life roles and environments, helping retirees to adjust and maintain their well-being despite the challenges associated with ageing and relocation. Migrated retirees often face language barriers, which limit their social interactions and this can lead to feelings of isolation (Haas, 2013). To enhance occupational engagement and connect with the local community, learning the language of the retirement country is important. Feeling connected and purposeful also makes people happier and improves their life quality (Haim-Litevsky et al., 2023). This is important for retirees, as it helps them enjoy their retirement despite the challenges of adapting to a different culture and environment. Experiences involving retirement and migration, as co-occurring, can be examined through these lenses. The concept of occupational adaptation suggests that successful adjustment in retirement and migration involves continuous interaction between the person and their new environment, where meaningful occupation plays a central role in achieving a sense of mastery and satisfaction (Schkade and Schultz, 1992). However, existing literature predominantly focuses on the perspective of the migration transition.

Moreover, a concern among migrated retirees was that they were seen as foreigners in their new communities (Ashton et al., 2019). Being seen as a foreigner could affect their social roles and occupational engagements, as being seen as a foreigner might limit opportunities for deeper community involvement and fulfilling social participation. Occupational science recognises the importance of these social roles and the sense of identity they can bring (Gallagher et al., 2015), especially in retirement, where individuals are navigating the transition from their work life to a new phase of life. Individuals frequently shape their identity and sense of purpose through their professional roles. However, retirement necessitates a shift to new roles and activities, effectively filling the gap left by the absence of work. This transition is crucial for retirees’ well-being, as it facilitates the development of a renewed sense of identity and purpose beyond their careers, ensuring they stay engaged and connected.

Studies exploring the healthcare experiences of migrated retirees show varying levels of satisfaction (Haas, 2013; Sloane et al., 2013). Studies concur that financial constraints or considerations can affect migrated retirees’ ability to access healthcare (Blaakilde, 2015). In contrast, Bender et al. (2018) state that healthcare quality and availability are significant factors in choosing a retirement destination. Interestingly, while included studies did not often report healthcare as a decisive factor, other research highlighted it as an important factor when considering retirement destinations (Marquez et al., 2024). Özyurt (2023) found that retiree migrants improved their well-being by spending more time outside.

Studying retirement migration as two co-occurring occupational transitions offers a more comprehensive understanding of how individuals adapt to new life circumstances. Retirement signifies a transition away from structured work roles, altering daily routines and identity, while migration is a transition that disrupts established environments, social networks and cultural standards. When these transitions co-occur, they create unique challenges and opportunities for individuals as they navigate new roles, routines and occupations in unfamiliar settings.

### Limitations

The initial search revealed a wide range of studies across multiple disciplines, such as tourism, social sciences and economics, each with its own definition of retirement migration. This systematic literature review focused on when retirement meant a complete cessation of work and migration involved moving permanently beyond the home country’s borders. This specific focus was chosen because the nature of the transition into retirement could significantly differ for those partially engaged in work. This also accounted for the variations in retirement migration, such as expatriates returning home for retirement and migrating seasonally. Studies that included internal migration were excluded due to their lack of significant environmental change, such as in culture or language, as this would affect how the migration transition was experienced. The lack of consistent and standardised definitions for retirement migration across various studies presented a challenge in drawing clear and definitive conclusions. The absence of a standardised definition of retirement migration complicates the design of studies and the comparison of findings across different contexts. This could lead to inconsistent results, making it difficult to draw a broader conclusion about how participants experience retirement migration. This could make it difficult for healthcare professionals to provide services effectively as they cannot meet the retirees’ needs. This could also affect policies’ development without a clear understanding of retirement migration. Further research should state clearly the definitions of retirement and migration. Studying retirement migration as two simultaneous and dynamic, co-occurring transitions could offer a better understanding of how individuals experience retirement migration.

The exclusion of articles not written in English and the limitations to exclude publications prior to the year 2000 may have prevented other relevant material from being included. The small number of included articles is acknowledged; however, this is the only material that meets the criteria. It is also noted that several of the included studies explicitly stated their findings were not intended to be generalisable, often due to small sample sizes or unique contextual factors. This reservation is a common feature of qualitative research, which aims to provide in-depth insight in experiences and processes.

## Conclusion

Retirement migration involves navigating new cultural, social and healthcare environments to maintain competence and mastery in daily activities, which is driven by and significantly impacts their economic conditions, integration and overall well-being. Factors like lower living costs and climates are crucial in the decision-making process. From the beginning, these factors make a more relaxed leisure life possible, a sort of prolonged vacation. However, as time goes by, other factors towards community involvement, social participation and engagement become more prominent. This process would be very interesting for further studies from an occupational perspective.

This evidence can guide the development of policies and healthcare services for migrated retirees. Occupational therapists should understand the diverse needs and experiences of migrated retirees. Recognising their motives, challenges and the benefits they seek in migrating can inform more effective support and integration strategies in their new communities.

Further research is warranted to understand the complexities of retirement migration transitions better. This includes exploring the occupational and social challenges faced by retirees. Such research is essential for creating tailored policies and services that address the unique needs of migrated retirees, ultimately enhancing their quality of life during this major life transition. As existing literature predominantly emphasises the migration transition, the retirement aspect, specifically how people shape their occupational identity, routines and sense of purpose, remains underexplored. To delve deeper into this topic, exploring retirement migration as a co-occurring occupational transition presents an opportunity for Occupational Science to add knowledge on this phenomenon. Future studies could investigate how retiree migrants navigate the loss or change of work-related roles, establish new occupations in unfamiliar environments and manage challenges such as visa restrictions or social isolation. Research can also examine how individual values, culture and life history shape their occupational choices in retirement migration. Additionally, longitudinal studies could explore the potential role of occupational therapy in supporting adaptation and well-being throughout the migration journey.

**Key findings**

- Economic factors and climate strongly influence retirement migration, offering better engagement opportunities.
- Community involvement is vital for retirees to feel belonging and contribute to their new country.

**What the study has added**

This study contributes insights into the occupational, social and well-being aspects of retirement migration, highlighting the importance of community integration and distinguishing types of retirement migration in research and policy.
